# Neuropeptides in the cerebral ganglia of the mud crab, *Scylla paramamosain*: transcriptomic analysis and expression profiles during vitellogenesis

**DOI:** 10.1038/srep17055

**Published:** 2015-11-23

**Authors:** Chenchang Bao, Yanan Yang, Huiyang Huang, Haihui Ye

**Affiliations:** 1 College of Ocean and Earth Sciences, Xiamen University, Xiamen 361102, China; 2 Collaborative Innovation Center for Development and Utilization of Marine Biological Resources, Xiamen 361102, China; 3 State Key Laboratory of Marine Environmental Science, Xiamen University, Xiamen 361102, China

## Abstract

Neuropeptides play a critical role in regulating animal reproduction. In vertebrates, GnRH, GnIH and kisspeptin are the key neuropeptide hormones of the reproductive axis, however, the reproductive axis for invertebrates is vague. Knowledge on ovarian development of the mud crab, *Scylla paramamosain*, is critical for aquaculture and resources management of the commercially important species. This study employed Illumina sequencing, reverse transcription-polymerase chain reaction and quantitative real-time PCR techniques to identify neuropeptides that may be involved in ovarian development of *S. paramamosain*. A total of 32 neuropeptide transcripts from two dozen neuropeptide families, 100 distinct mature peptides were predicted from the transcriptome data of female *S. paramamosain* cerebral ganglia. Among them, two families, *i.e.* GSEFLamide and WXXXRamide, were first identified from the cerebral ganglia of crustaceans. Of these neuropeptides, 21 transcripts of interest were selected for further confirmation and all of them were detected in the cerebral ganglia, as well as in other nervous tissues and the ovary. Most of them also had differential expression in the cerebral ganglia during various vitellogenic stages, suggesting their likely involvement in regulating vitellogenesis and ovarian maturation. Overall, these findings provide an important basis for subsequent studies on peptide function in reproduction of *S. paramamosain*.

Neuropeptides are ubiquitous in the nervous system of the animal kingdom from cnidarians to human^1^. By binding to receptors on target organs, neuropeptides exhibit autocrine, paracrine and hormonal effects, and often act as neurotransmitters or neuromodulators in the nervous system^2^. In general, neuropeptides are produced from a precursor (preproneuropeptide) and become biologically active after post-translational modifications^3^. The mature peptides are often short and of small size, and play key roles in many physiological processes, such as reproduction, metabolism, growth and locomotion^3^.

In vertebrates, reproduction is under the control of the key hormones of the hypothalamic-pituitary-gonadal (HPG) axis^4^,^5^,^6^. The hypothalamus is capable of sensing external and internal inputs, and accordingly regulates the biosynthesis and secretion of gonadotropin-releasing hormone (GnRH), a decapeptide with conserved motif, which is secreted via the hypophysial portal system to stimulate synthesis and secretion of the gonadotropins, *i.e.* luteinizing hormone (LH) and follicle-stimulating hormone (FSH)^4^. LH and FSH then act on the gonads to stimulate the production of gametes, steroid and peptide hormones^5^. Moreover, the recent discovery of two key neuropeptides, gonadotrophin-inhibitory hormone (GnIH) and kisspeptin enrich the vertebrate reproduction axis^6^. In 2000, GnIH was isolated from the brain of Japanese quail, *Coturnix japonica*, using high-performance liquid chromatography and a competitive enzyme-linked immunosorbent assay, and has been shown to inhibit gonadotropin release^7^. In 2003, the role of kisspeptin in regulating reproductive function was first reported^8^. In contrast to GnIH, kisspeptin has been shown to stimulate GnRH and gonadotropin secretions in mammalians^9^ and teleosts^10^, respectively. However, no similar peptides have been identified in crustaceans to date.

Crustaceans have served as important models for neuropeptides study in the past half century^11^. For example, crustacean X-organ-sinus gland (XO-SG) complex system in the eyestalk was the first formal demonstration of neurosecretion^12^. The first invertebrate neuropeptide, red pigment concentrating hormone (RPCH), was also fully characterized from a shrimp species^13^. With the advent of new methodologies, such as genome and transcriptome mining and mass spectrometry, more than two dozen neuropeptide families have been characterized from various crustacean species^11^. Because of its historical status and ubiquitous conservative property, XO-SG complex become a major well studied neuroendocrine organ and their importance in the field of neuroendocrinology is undisputable. In crustaceans, the studies of reproduction also focused on XO-SG complex neuropeptides, *i.e.* crustacean hyperglycaemic hormone (CHH), moult-inhibiting hormone (MIH), gonad/vitellogenesis-inhibiting hormone (GIH/VIH) and mandibular organ-inhibiting hormone (MOIH)^14^. Compared with XO-SG, however, the neuroendocrine status of cerebral ganglia have somewhat been neglected in crustaceans. In vertebrates, the brain is the center of neural and humoral regulation. The cerebral ganglia of crustaceans, also known as supraesophageal ganglia, are consisted of protocerebrum, deutocerebrum, and tritocerebrum in decapods^15^. Not only do they play a role of neuromodulation, but also control the release of hormones such as gonad stimulating hormone^16^,^17^. In recent years, many neuropeptides, *i.e.* A-type allatostatin (AST-A), B-type allatostatin (AST-B), C-type allatostatin (AST-C), neuropeptide F(NPF), short neuropeptide F (sNPF), myosuppressin, sulfakinin, neuroparsin (NP), crustacean hyperglycemic hormone (CHH), orcokinin, crustacean cardioactive peptide (CCAP), diuretic hormone 31 (DH31), eclosion hormone (EH), HIGSLYRamide, kinin, pyrokinin, pigment-dispersing hormone (PDH), red pigment-concentrating hormone (RPCH), SIFamide (SIF), tachykinin (TK) and vasotocin-neurophysin, have been found in the cerebral ganglia of various crustaceans via transcriptomics and mass spectrometry^18^,^19^,^20^,^21^. We speculate that there are also some neuropeptides to be mined in the cerebral ganglia of crustaceans, and further research on their functions is warranted.

It is significant to study crustacean reproductive endocrinology for developing aquaculture techniques and understanding its basic biology. The mud crab, *Scylla paramamosain*, is a commercially important crab species in the Indo-Pacifics^22^. Although large-scale next-generation sequencing research for the muscle, hepatopancreas, eyestalk, haemolymph and gonads of *S. paramamosain* had been reported^23^,^24^, more need to be done on the analysis of peptidome, particularly on the reproductive function of the enormous neuropeptide families from the cerebral ganglia. This paper reports results of employing techniques of next-generation sequencing (Illumina sequencing), reverse transcription-polymerase chain reaction (RT-PCR) and quantitative real-time PCR (qRT-PCR) to exploit potential neuropeptides from the cerebral ganglia and confirm their roles during vitellogenesis of *S. paramamosain*.

## Results

### Neuropeptides discovery by transcriptome mining: bioinformatics analyses and peptide prediction

Large-scale peptidome for *S. paramamosain* was predicted using the Illumina sequencing. In the absence of genome, transcriptome mining is a powerful tool for neuropeptides discovery of *S. paramamosain*. One hundred distinct mature peptides were identified in the cerebral ganglia of female *S. paramamosain*. 32 transcripts encoding neuropeptide precursors (23 complete and 9 partial) were identified, with a total of 100 distinct mature peptides predicted from this transcriptome data (Table 1, Supplementary file and Supplementary Table S1). The deduced *S. paramamosain* peptides include isoforms of AST-A, AST-B, AST-C, NPF, sNPF, FLRFamide, myosuppressin, sulfakinin, NP, CHH, orcokinin, CCAP, DH31, EH, GSEFLamide, HIGSLYRamide, kinin, pyrokinin, PDH, RPCH, SIF, TK, WXXXRamide, vasotocin-neurophysin as well as a myriad of linker/precursor-related peptides (Table 1 and Supplementary file). Among these peptides, GSEFLamide and WXXXRamide were firstly identified from the cerebral ganglia of a crustacean.

#### AST-A

**Figure 1:**
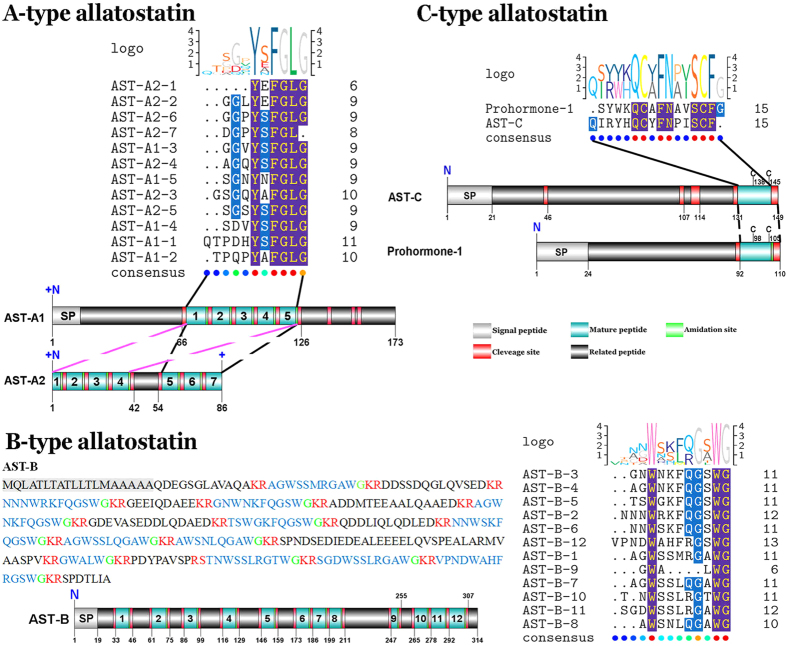
Identification and characterization of *S. paramamosain* ASTs peptides. (A-type allatostatin) Schematic representation of two AST-A precursors and their 12 mature peptides comparative sequence alignments in *S. paramamosain*, sequence logo is shown above alignments. (B-type allatostatin) Amino acid sequences and schematic representation of AST-B precursor in *S. paramamosain*, logo is shown above comparative sequence alignments of AST-B peptides. (C-type allatostatin) Schematic representation of AST-C and prohormone-1 precursors in *S. paramamosain* and their mature peptides comparative sequence alignments, sequence logo is shown above alignments.

Two transcripts were identified to putatively encode partial AST-A precursors representing the C-terminus (AST-A1) and the middle region (AST-A2), with 173 and 86 amino acids (aa), respectively (Supplementary file and Fig. 1). The precursor AST-A1 has a part of the predicted signal peptide (15aa), followed by 5 predicted peptides, and separated by dibasic cleavage sites (Fig. 1). The AST-A2 contains 7 predicted peptides, the first predicted peptide (AST-A2-1) misses N-terminus and the last predicted peptide (AST-A2-7) lacks a glycine, which is implicated in amidation of the peptide (Fig. 1). Sequences of 12 predicted mature peptides containing the signature with X**Y**X**FGLamide** (where X represent variable residues) highly conserved motif at the C-terminus (Fig. 1) of AST-As. Each of the predicted mature peptides is inequable.

#### AST-B

A single transcript in the transcriptome assembly putatively encoded the complete precursor of AST-B with 314aa, starting with a predicted signal peptide of 19aa, followed by 12 predicted mature peptides, separated by dibasic cleavage sites (Fig. 1). The 12 predicted peptides are 5–12aa in length with X**W**XXXX**G**X**Wamide** conserved motif (Fig. 1).

#### AST-C

The predicted sequence of AST-C precursor is composed of 149aa, starting with a 21aa signal peptide (Supplementary file and Fig. 1). The mature peptide has a typical structure characterized by the full length of 15aa, the presence of a pyroglutamine blocked N-terminus, the unamidated C-terminal motif -PISCF, and a disulfide bridge between the cysteine residues located at positions 7 and 14 (Fig. 1)^11^. Prohormone-1 precursor, an AST-C-like peptide precursor, was also identified in *S. paramamosain* with 110aa and a signal peptide of 24 residues (Supplementary file and Fig. 1). The prohormone-1, SYWK**QC**A**FN**AV**SCF**amide, shares a conserved motif with the AST-C (Fig. 1I).

#### NPF

**Figure 2:**
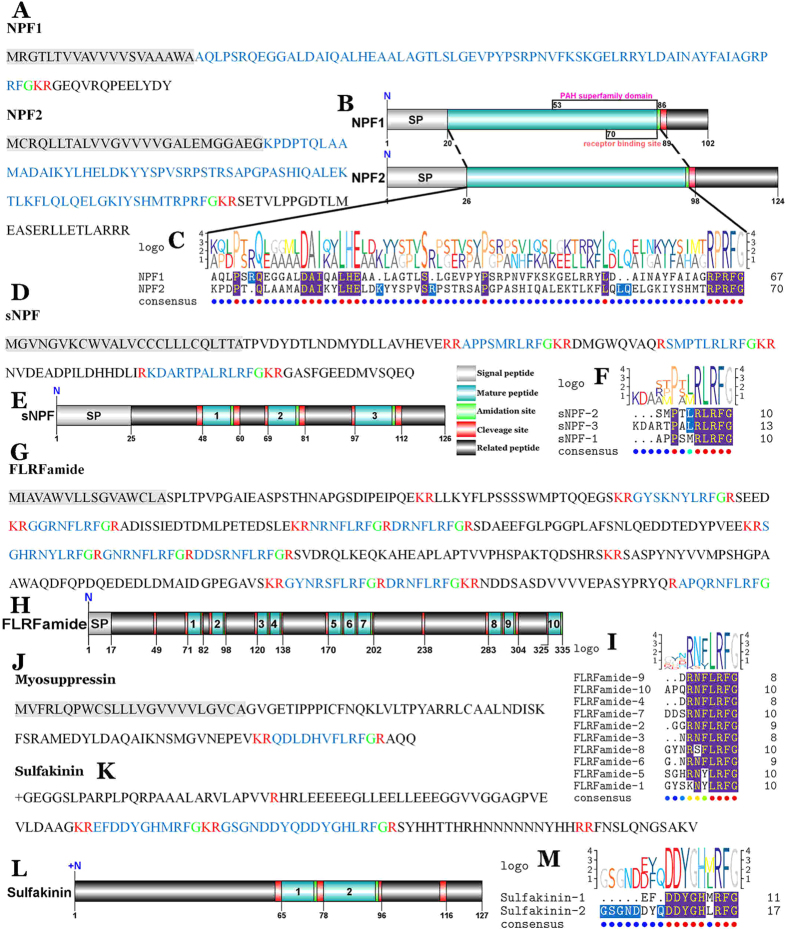
Identification and characterization of *S. paramamosain* RFamide peptides. (**A,D,G,J,K**) Amino acid sequences of RFamide precursors. (**B,E,H,L**) Schematics showing organization of RFamide precursors identified in *S. paramamosain*. (**C,F,I**) Comparative sequence alignments of RFamide peptides, sequence logos are shown above alignments.

Two transcripts were identified to putatively encode complete RPRFamide peptide precursors with 102 and 124aa, including a predicted signal peptide of 20 and 26aa (Fig. 2A,B). The mature peptides are encoded immediately after the signal peptide and terminate with a glycine, which permits amidation of the C-terminal (Fig. 2A,B). The predicted 66aa mature peptide, NPF1, with a pancreatic hormone (PAH) superfamily domain and a receptor binding site (Fig. 2B), has best BLAST match with the mollusk neuropeptide Y (NPY) (Table 1). NPF2, the predicted mature peptides with 69aa, showed 73% identity with the NPF II from the Pacific white shrimp, *Litopenaeus vannamei* (Table 1).

#### sNPF

One transcript was identified to putatively encode a complete sNPF precursor with 126aa, starting with a 25aa signal peptide, followed by three predicted peptides (Fig. 2D,E), and the three predicted peptides are 9–12aa in length with X**P**X**RLRFamide** conserved motif (Fig. 2F). From the LOGO of NPFs and sNPF, we find that they have conserved C-terminal: **R**X**RFamide**, but the sNPF is not a short alternative spliced form of NPFs, as there are many important differences in the precursors (Fig. 2A,D).

#### FLRFamide

The nucleotide sequence produced from the transcriptome data covered 1888 bp putatively coding for a complete FLRFamide precursor with 335aa (Table 1). The precursor starting with a signal peptide of 17aa, followed by 10 predicted mature peptides (Fig. 2G,H). The mature peptides are 7–9aa in length with R/K-N/S-F/Y-LRFamide conserved motif (Fig. 2I).

#### Myosuppressin

The predicted sequence of the myosuppressin precursor is composed of 100aa with a signal peptide of 24aa (Fig. 2J). The predicted mature peptide is a 10aa peptide, blocked at both ends. The most common form of this peptide is: pQDLDHVFLRFamide^11^ (Fig. 2J).

#### Sulfakinin

The putative transcript encoded a partial C-terminus of sulfakinin precursor with 128aa (Fig. 2K,L). Two putative sulfakinin peptides, EFDDYGHMRFamide and GSGNDDYQDDYGHLRFamide, were separated by carboxy-peptidase cleavage sites (Fig. 2K,L) and they have conserved C-terminal: **DDYGH**X**RFamide** (Fig. 2M).

#### NP

**Figure 3:**
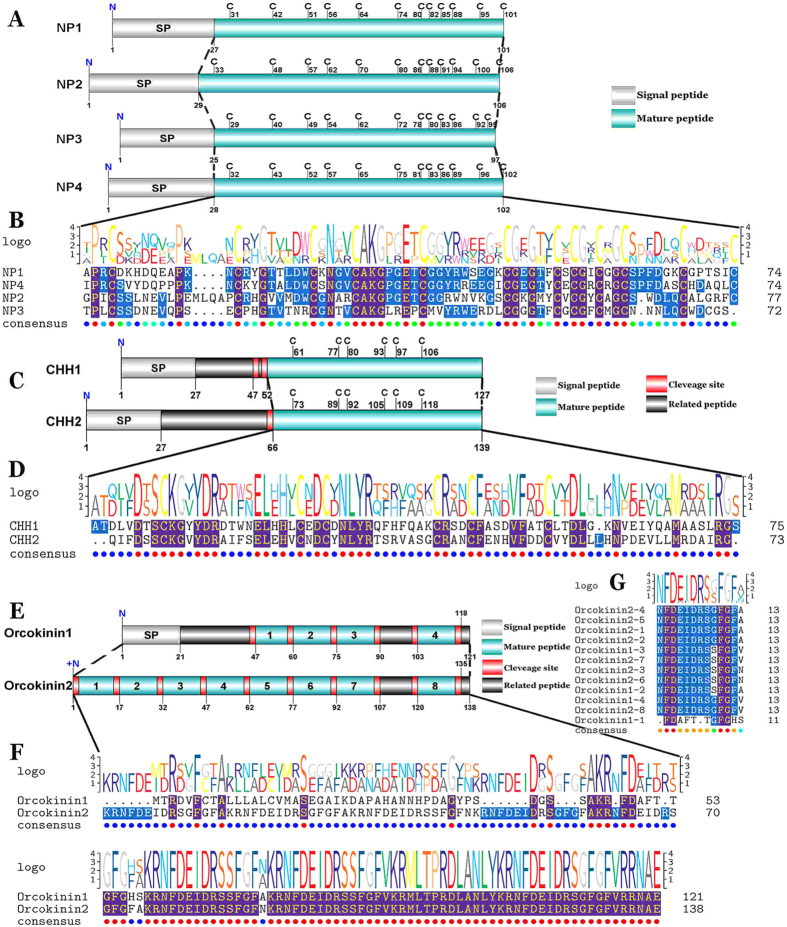
Identification and characterization of *S. paramamosain* NP, CHH and orcokinin. (**A,C,E**) Schematics showing NP, CHH and orcokinin precursors identified in *S. paramamosain*. (**B,D,G**) Comparative sequence alignment of NP, CHH and orcokinin mature peptides, sequence logos are shown above alignments. (**F**) Comparative sequence alignments of orcokinin precursors, sequence logo is shown above alignments.

Four transcripts were identified to putatively encode complete NP precursors with 97–106aa. All four complete sequences start with a predicted signal peptide of 25–29aa. The predicted mature peptides of 72–77aa are encoded immediately after the signal peptide (Supplementary file and Fig. 3A). 12 cysteine residues of all four mature peptides are aligned, except for the last cysteine residue of NP3 (Fig. 3B).

#### CHH

Two transcripts were identified to putatively encode complete CHH precursors with 127 and 139aa (Supplementary file and Fig. 3C). CHH1 precursor could be partitioned into a signal peptide of 27aa, followed by a 20aa CHH-PRP (CHH precursor-related peptide) and finally a mature peptide of 75aa (Fig. 3C). CHH1 is a novel neuropeptide found in the mud crab. However, CHH2 is identical with our previous study from the eyestalk of *S. paramamosain*^25^, in that it starts with a 27aa signal peptide, followed by a 37aa CHH-PRP and a mature peptide of 73aa, separated by dibasic cleavage site (Fig. 3C). This is similar with both of CHH have six cysteine residues located in the mature peptides and the six cysteines are aligned (Fig. 3D).

#### Orcokinin

Two transcripts were identified to putatively encode a complete (orcokinin1) and a partial C-terminus (orcokinin2) orcokinin precursor with 121 and 138aa, respectively (Supplementary file and Fig. 3E). Orcokinin1 has a predicted signal peptide of 21aa and 4 predicted mature peptides, and the orcokinin1 C-terminus with sequence identity to the C-terminus of orcokinin2 (Fig. 3F). Orcokinin2 has 8 predicted mature peptides, but it lacks the signal peptide (Fig. 3E). In the 12 predicted mature peptides, 11 peptides are 13aa in length with **NFDEIDRS-G/S-FGF**X conserved motif, but the orcokinin1-1 has 11aa with structure: FDAFTTGFGHS (Fig. 3G), which was also named as orcomyotropin because it could enhance hindgut contractility^11^.

#### CCAP

**Figure 4:**
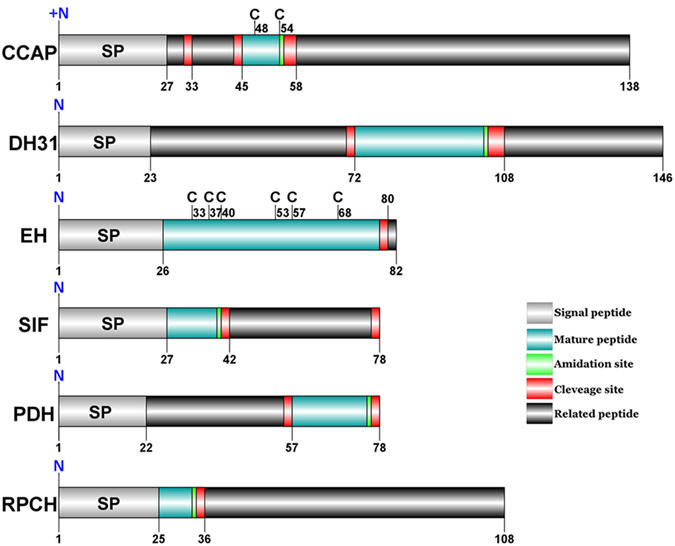
Schematic representation of *S. paramamosain* CCAP, DH31, EH, SIF, PDH and RPCH neuropeptides. Schematic showing CCAP, DH31, EH, SIF, PDH and RPCH precursors identified in *S. paramamosain*.

The predicted sequence of the CCAP precursor is composed of 138aa with a predicted signal peptide of 27aa (Supplementary file and Fig. 4). Two cysteines located in the CCAP mature peptide, which is a C-terminal amidated nonapeptide start at position 46 and finished at position 55 (Fig. 4). Four CCAP-PRPs (CCAP precursor-related peptide) are present in the propeptide, two upstream of CCAP and two downstream (Supplementary file and Fig. 4).

#### DH31

A DH31 transcript was present in the assembly of *S. paramamosain*. The precursor putatively comprised 146aa with a 23aa predicted signal peptide and the 31aa C-terminal amidated DH31 mature peptide is released using dibasic cleavage sites (Supplementary file and Fig. 4).

#### EH

One transcript was identified to putatively encode a complete EH precursor comprising 82 residues with a predicted signal peptide of 26aa (Supplementary fileand Fig. 4). The remanent 56aa propeptide, containing a dibasic cleavage site, can release a 52aa EH mature peptide with 6 cysteines (Fig. 4).

#### GSEFLamide

**Figure 5:**
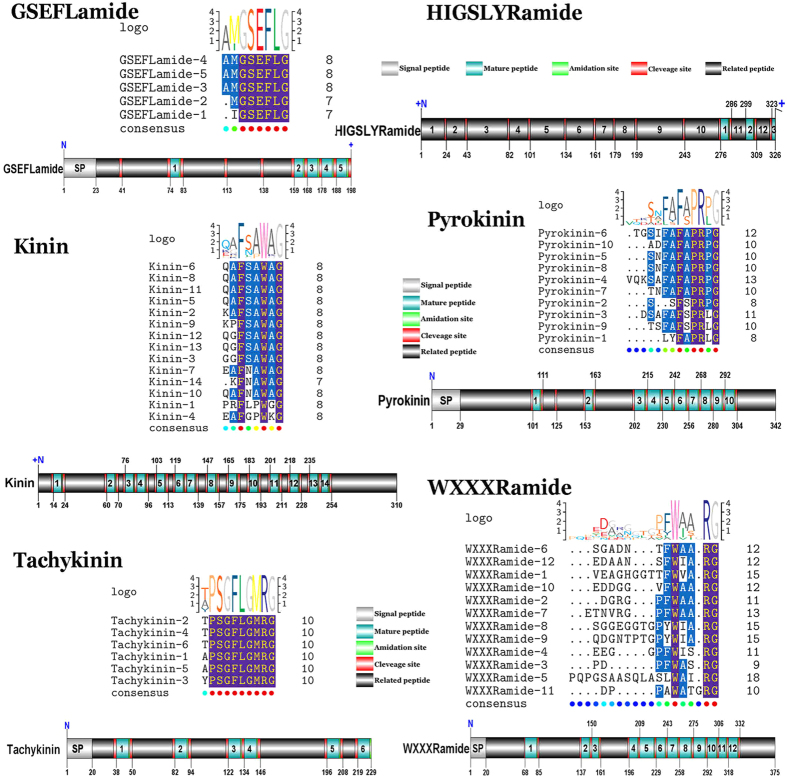
Identification and characterization of GSEFLamide, HIGSLYRamide, kinin, pyrokinin, tachykinin and WXXXRamide in *S. paramamosain*. Schematic showing GSEFLamide, HIGSLYRamide, kinin, pyrokinin, tachykinin and WXXXRamide precursors identified in *S. paramamosain*. A sequence logo is shown above alignments, where the height of each letter is proportional to the observed frequency of the corresponding amino acid in the alignment column.

The putative sequence encoded a partial N-terminus of GSEFLamide precursor with 198aa (Supplementary file and Fig. 5). The partial precursor starts with a 23aa predicted signal peptide, followed by 5 predicted mature peptides, separated by dibasic cleavage sites (Fig. 5). The 5 predicted mature peptides are 6–7aa in length with X**GSEFLamide** conserved motif (Fig. 5). The GSEFLamide neuropeptide family had been described from the analysis of transcriptome of the intertidal copepod, *Tigriopus californicus*^26^ and the Pacific white shrimp, *L. vannamei*^27^, however, the GSEFLamide neuropeptide was first discovered from the cerebral ganglia of crustacean in this study.

#### HIGSLYRamide

The sequence of the putative HIGSLYRamide precursor in the *S. paramamosain* transcriptome is part of middle region with 326aa (Supplementary file and Fig. 5). Regardlessly, 14 dibasic sites are contained within the sequence, putatively liberating as many as 15 peptides (Fig. 5). Two of these peptides both have the structure: HIGSLYRamide (Fig. 5), and the “HIG” peptide of 3 amino acids appears to be a part of HIGSLYRamide, is predicted from the incomplete N-terminus (Fig. 5). 12 other peptides were identified as precursor-related peptide (Fig. 5).

#### Kinin

One transcript was identified to putatively encode 310aa of the C-terminus of the kinin precursor (Supplementary file and Fig. 5). The precursor has 14 predicted mature peptides, separated by dibasic cleavage sites (Fig. 5). The 14 predicted peptides are 6–7aa in length with X**F**X-**A/P**-**W**X**amide** conserved motif, and four peptides of them have the same structure: QAFNAWAamide (Fig. 5).

#### Pyrokinin

A complete pyrokinin precursor of 342aa, starting with a predicted signal peptide of 29aa, followed by 10 predicted mature peptides, and separated by dibasic cleavage sites (Supplementary file and Fig. 5). The 10 predicted pyrokinin peptides are 7–12aa in length with a common conserved motif: F-A/S-PR-P/Lamide (Fig. 5). The motif is similar to a conserved C-terminal pentapeptide, FXPRLamide which belong to the pyrokinin/pheromone biosynthesis activating neuropeptide (PBAN) peptide family in insects^28^.

#### PDH

The putative PDH precursor is comprised of 78aa, starting with a predicted signal peptide of 22aa, and the C-terminal amidated PDH mature peptide was released by two dibasic cleavage sites (Supplementary file and Fig. 4). PDH possess two types (α-PDH and β-PDH), and share a conserved structure with a mature peptide of around 18aa^29^. Meanwhile, one isoform of β-PDH had been cloned from the eyestalks of *S. paramamosain* in our previous study^29^.

#### RPCH

The predicted sequence of the RPCH precursor is composed of 108aa, starting with a predicted signal peptide of 25aa, followed by an octapeptide immediately, pQLNFSPGWamide, which is blocked at both ends (Supplementary file and Fig. 4). The sequence of RPCH precursor in this study is identical to the RPCH precursor reported from another mud crab, *Scylla olivacea*^30^.

#### SIF

In the transcriptome assembly, a single transcript putatively encoded the complete SIF precursor with 78aa, starting with a predicted signal peptide of 27aa, and the predicted mature peptide, GYRKPPFNGSIFamide, is encoded immediately after the signal peptide (Supplementary file and Fig. 4). The SIF precursor has a high homology (93% identity) with SIF of the Jonah crab, *Cancer borealis* (Table 1). Besides the SIF precursor, SIF mature peptide exhibit conserved structure: X**YRKPPFNGSIFamide**^11^.

#### TK

One transcript was identified to putatively encode a complete TK precursor with 229aa, starting with a predicted signal peptide of 20aa, followed by 6 predicted TK peptides of 9aa, separated by carboxy-peptidase cleavage sites (Supplementary file and Fig. 5). All six TK peptides have high similarity with conserved motif: X**PSGFLGMRamide** (Fig. 5), which belongs to the TK family possessing the C-terminal motif -**F**X**G**X**Ramide**, is broadly conserved in invertebrates^11^.

#### WXXXRamide

During the search for TK precursor, one transcript encoding a complete new neuropeptide family was discovered. Complete 375aa open reading frame (ORF) of the new neuropeptide family precursor starting with a predicted signal peptide of 20aa, followed by 12 predicted mature peptides, separated by carboxyl-peptidase cleavage sites (Supplementary file and Fig. 5). 12 mature peptides are 8–14aa in length with **W**XXX_n_**Ramide** (where Xn represents a glycine or nothing) conserved motif (Fig. 5).

#### Vasotocin-neurophysin

**Figure 6:**
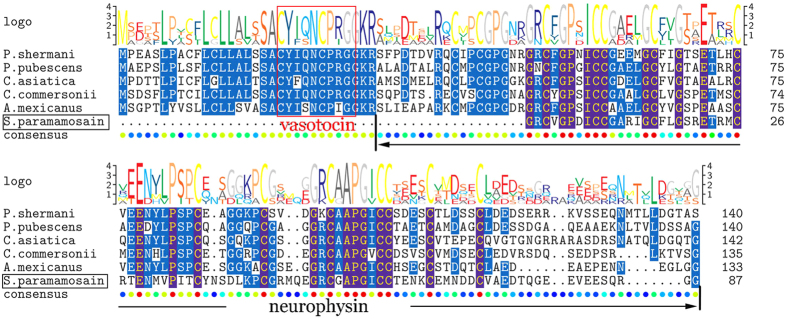
Comparative sequence alignment of vasotocin-neurophysin in different animals. Comparative sequence alignment of vasotocin-neurophysin precursors for *S. paramamosain* with *Plethodon shermani*, *Picoides pubescens*, *Chrysochloris asiatica*, *Catostomus commersonii* and *Astyanax mexicanus*. Sequence logo is shown above alignments.

A complete vasotocin-neurophysin precursor starts with a signal peptide, then following a mature vasotocin peptide and a neurophysin domain with 14 cysteine residues (Fig. 6). Nevertheless, only a portion of vasotocin-neurophysin precursor was identified to putatively encode only a part of neurophysin with 12 cysteines of 104aa and no signal peptide and mature vasotocin peptide or any dibasic sites were putative (Supplementary file and Fig. 6). There is little information about vasotocin peptide in arthropoda^31^. In crustaceans, moreover, the vasotocin peptide and its receptor had only been described in the water flea, *Daphnia pulex*^32^ and the giant freshwater prawn, *Macrobrachium rosenbergii*^21^, but their function was unclear.

#### Neuropeptide verification by RT-PCR: tissue distribution

**Figure 7:**
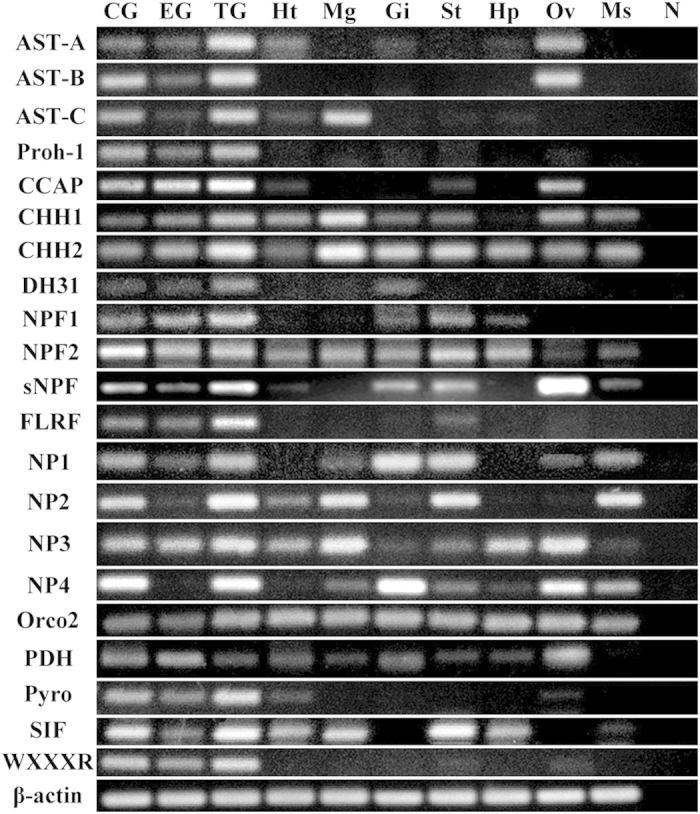
Tissue distribution of *S. paramamosain* neuropeptide transcripts by RT-PCR. 21 neuropeptide transcripts were detected by RT-PCR in ten tissues of *S. paramamosain*: CG, cerebral ganglia; EG, eyestalk ganglia; TG, thoracic ganglia; Ht, heart; Mg, midgut; Gi, gill; St, stomach; Hp, hepatopancreas; Ov, ovary; Ms, muscle; N, negative control (representing a PCR reaction performed without adding template). β-actin as the reference gene.

To confirm the neuropeptide transcripts were actually synthesized in the cerebral ganglia of *S. paramamosain* and to check whether these genes are also expressed in other tissues, RT-PCR was utilized to detect their expression in ten tissues of *S. paramamosain*. 21 transcripts of interest were detected in various tissues (Fig. 7). All of them were detected in the nervous system (cerebral ganglia, eyestalk ganglia and thoracic ganglia), but also widely distributed in other tissues (Fig. 7). Remarkably, most of them (16 transcripts) were also detected in the ovary (Fig. 7).

### Neuropeptide verification by qRT-PCR: the expression profiles during vitellogenesis

**Figure 8:**
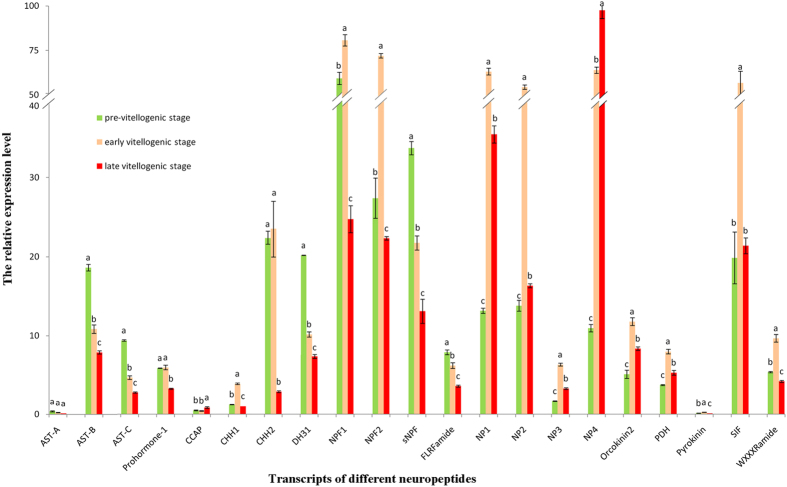
Expression profiles of *S. paramamosain* neuropeptide transcripts by qRT-PCR. The expression levels of 21 neuropeptide transcripts in the cerebral ganglia from *S. paramamosain* at different vitellogenic stages. β-actin as internal control gene. Each bar represents the mean value of quintuplicate ± SD. Different letters at the top of bars indicate significant differences between various vitellogenic stages, “a” represents the highest relative expression level of neuropeptide transcript, “b” represents neuropeptide transcript had significantly lower relative expression level than “a” but significant higher expression level than “c”, “c” represents neuropeptide transcript had significantly lower relative expression level than “a” and “b”, respectively (P < 0.05, one-way ANOVA followed by Duncan’s multiple range tests, n = 5).

To investigate the potential involvement of neuropeptides in reproduction of *S. paramamosain*, neuropeptide genes expression levels in the cerebral ganglia of female crabs during three ovarian development stages (pre-vitellogenic, early vitellogenic and late vitellogenic stage) were determined. Due to no replicate samples for Illumina sequencing, the values of RPKM (number of reads mapped to the transcript per kilobase per million reads in the total library) were only relative^33^. Thus, it was necessary to verify the expression levels of neuropeptide genes by qRT-PCR. Twenty-one predicted neuropeptide genes were subjected to qRT-PCR. Most of them were differentially expressed during vitellogenesis: *ast-b* (B-type allatostatin), *ast-c* (C-type allatostatin), *dh31* (diuretic hormone 31), *flrf* (FLRFamide) and *snpf* (short neuropeptide F) were continuous significantly down-regulated during vitellogenesis. *chh1* (CHH1), *npf1* (neuropeptide F1), *npf2* (neuropeptide F2), *np1* (neuroparsin1), *np2* (neuroparsin2), *np3* (neuroparsin3), *orco2* (orcokinin2), *pdh* (pigment-dispersing hormone), *pk* (pyrokinin), *sif* (SIFamide) and *wxxxr* (WXXXRamide) were significantly up-regulated at the early vitellogenic stage compared to the pre-vitellogenic stage but significantly down-regulated at the late vitellogenic stage while *proh1* (prohormone-1) and *chh2* (CHH2) were specifically down-regulated at the late vitellogenic stage compared to other stages. On the contrary, *ccap* (crustacean cardioactive peptide) was specifically up-regulated at the late vitellogenic stage compared to other stages. Unlike other three *nps* (neuroparsins), *np4* (neuroparsin4) was up-regulated continuously during vitellogenesis. Finally, *ast-a* (A-type allatostatin) showed no significant change in expression during the three vitellogenic stages although had the trend similar to *ast-b* and *ast-c* (Fig. 8).

## Discussion

The present study represents the first large-scale RNA sequencing for the cerebral ganglia of the mud crab *S. paramamosain*. One hundred distinct mature peptides from 32 neuropeptide precursors were predicted in transcriptome data and 21 of these neuropeptide genes were detected in different tissues by RT-PCR. The fact that all of the 21 neuropeptide genes were detected in the cerebral ganglia (Fig. 7) demonstrates that the neuropeptide sequences from transcriptome data are reliable and confirm that these neuropeptide transcripts were synthesized in the cerebral ganglia of *S. paramamosain*. Furthermore, these neuropeptide genes were also widely expressed in various tissues including ovary (Fig. 7), suggesting that their function as neuroendocrine in the nervous system, as well as autocrines/paracrines in the non-nervous system.

During oocytes’ development of oviparous animals, the accumulation of yolk in oocytes is essential for proper embryonic development after fertilization and is therefore, a key process in successful reproduction^34^. In *S. paramamosain*, the onset of vitellogenesis is at the early vitellogenic stage, while the accumulation of yolk is mostly at the late vitellogenic stage^35^. Thus, the gene expression differences of the neuropeptides during vitellogenesis may indicate their involvement in vitellogenesis and ovarian maturation.

In insects, juvenile hormone (JH) is involved in the regulation of reproduction, and its synthesis is inhibited by allatostatins (**ASTs**), a pleiotropic neuropeptides^36^. There are evidences that Methyl farnesoate (MF), the unepoxidised form of JH III uniquely discovered in crustaceans, may act as crustacean JH and stimulated ovarian maturation in crustaceans^37^. Since the discovery of ASTs in *S. paramamosain*, exploring the relationship between ASTs and MF has been an interesting topic. In *S. paramamosain*, the expression levels of three types of ASTs continuously decreased during vitellogenesis (Fig. 8), indicating that they may be involved in similar physiological process as in insects. Considering the JH-inhibiting effects of ASTs in insects, the significant lower expression levels of ASTs detected at the late vitellogenic stage of *S. paramamosain* might reduce its suppressive effect on MF synthesis and hence stimulate ovarian maturation.

**NPF**, an invertebrate NPY-like family, shares similar sequences and likely the physiological functions as NPY^38^. In vertebrates, NPY is a 36aa peptide from the pancreatic polypeptide family, acts as the modulator between energy balance and reproduction^39^. Depending on the sex steroid milieu, NPY can promote or inhibit surges of LH, thus have contradictory effects on gonadotropins^40^,^41^. With higher circulating concentrations of estrogen, NPY had a stimulatory effect on GnRH release from rat median eminence *in vitro*^42^. Meanwhile, trNPF, a 9aa NPF, has been reported to stimulate oocyte maturation, food intake, weight increase and regulates male reproductive processes in adult desert locusts, *Schistocerca gregaria*^43^,^44^,^45^. To date, the reproductive function of NPF has been reported in *Schmidtea*, *Lymnaea*, *Aplysia* and *Drosophila*^38^. In crustaceans, many NPF peptides have recently been predicted via transcriptomics or molecular cloning and it has already been confirmed that NPF could stimulate food intake and growth in *L. vannamei*^46^. However, no information is currently available on their potential reproductive function in crustaceans. In this study, the high expression of NPFs at the early vitellogenic stage (Fig. 8) indicates that NPFs might be involved in stimulating food intake to supply energy for ovarian maturation.

**sNPF** is also classified as a NPY-like peptide, even though the precursors of NPF and sNPF are not likely to be very closely related. Experimental data demonstrated that in insects, the roles of sNPF are multiple, including regulating feeding, growth and metabolic homeostasis^38^. Moreover, a peptide signal pathway involving sNPF has been discovered in the corpora cardiaca–corpora allata of the silkworm, *Bombyx mori*^47^. It was found that the corpora cardiaca synthesized sNPF as a stage-specific suppressor for JH biosynthesis by the corpora allata^47^,^48^. Since sNPF was reported to have JH-inhibiting effects^47^,^48^, and the expression pattern of sNPF in our study is similar to that of ASTs (Fig. 8), we speculate that sNPF is closely related to ASTs and may suppress MF synthesis and leading to retard ovarian development. Similar to sNPF, **FLRFamide** have conserved C-terminal (-RFamide) and the expression level of their transcript decreased continuously during vitellogenesis (Fig. 8). FLRFamide have been well identified in crustaceans as powerful modulators of the cardiovascular system, the digestive tract and exoskeleton muscles^11^; thus, FLRFamide may be involved in organism activities and energy consumption during ovarian development in the mud crab *S. paramamosain*.

The first **NP** was identified from the migratory locust, *Locusta migratoria* as an anti-gonadotropic peptide that delayed vitellogenesis^49^. In *S. gregaria*, it was reported that the expression levels for two vitellogenin genes significantly increased upon a knock-down of the NPs, and NP dsRNA-treated females had significantly larger oocytes than those from the control^50^. Whereas in the mosquito, *Aedes aegypti*, ovary ecdysteroidogenic hormone (OEH), a NP-like peptide, was reportedly released from the cerebral ganglia into the haemolymph and stimulates the ovaries to secrete ecdysteroid hormones, which modulated yolk protein synthesis^51^. These studies suggest that NPs play an important role in regulating reproduction of insects. While many NP-like peptides have recently been predicted via transcriptome mining from crustaceans, little is known about their functional roles. The only exception is in the sand shrimp, *Metapenaeus ensis*^52^, the highest expression level of NP was recorded during the early gonadal maturation stage but decreased at the late maturation stage. Moreover, *in vivo* gene silencing of NP caused a significant decrease of vitellogenin transcript level in both hepatopancreas and ovary of *M.ensis*^52^. Similarly, the present study showed that NP1–3 had highest expression at the early vitellogenic stage and decreased sharply at the late vitellogenic stage in *S. paramamosain* (Fig. 8), indicating NP1-3 might be involved in stimulating vitellogenesis at the initial stage. In contrast to NP1-3, NP4 had highest expression level at the late vitellogenic stage during which yolk accumulation entering fast phase (Fig. 8), suggesting that NP4 might be involved in promoting yolk accumulation during late vitellogenesis.

Genes of **CHHs** and **PDH** had been cloned from the eyestalk of *S. paramamosain*^25^,^29^. The expression profiles of CHHs in the eyestalk ganglia suggested that it might be involved in promoting vitellogenesis in *S. paramamosain*^25^. Similarly, in the present study, higher expression of CHHs in the cerebral ganglia at the early vitellogenic stage (Fig. 8) suggests that CHHs as a promoting factor for vitellogenesis. On the other hand, the PDH expression maintained at high levels in the cerebral ganglia at the early vitellogenic stage but decreased significantly at the late vitellogenic stage (Fig. 8), which is similar to PDHs expression in the eyestalks and ovaries from a previous study^29^. PDH is known as a light adapting hormone controlling circadian rhythm^53^, whether it also plays a role in the process of crustacean reproduction requires further investigation.

**CCAP**, a cyclic nonapeptide, was originally identified from the European green crab, *Carcinus maenas* as a cardioaccelerator^54^ and reportedly could also modulate oviduct contraction in *L. migratoria*^55^. The presence of the CCAP receptor in the reproductive systems of male and female blood-sucking bug, *Rhodnius prolixus* indicated that CCAP may be involved in reproduction^56^. In this study, the significant up-regulation of transcript level of CCAP at the late vitellogenic stage (Fig. 8) is likely to accelerate heart contraction and haemolymph circulation^57^, which in turn could promote the transfer of exogenous vitellogenin into the ovary. **DH31** belongs to the DH family and has been shown to affect salt and water transportation by Malpighian tubules in several insects^58^. Thus, based on the continuous declining of its transcript level during vitellogenesis (Fig. 8), we speculate that DH31 may be involved in maintaining ionic homeostasis of haemolymph during ovarian development. **Orcokinin** was first isolated from the crayfish, *Orconectes limosus* as a myotropic peptide^59^ and bioactivities of orcokinin, *e.g.* increasing spontaneous hindgut contraction and modulating the output of the stomatogastric nervous system, has been demonstrated in several decapods^11^. However, no evidence was shown on their role on crustacean reproduction so far. In the present study, the highest expression of orcokinin2 was found at the early vitellogenic stage (Fig. 8), similarly in ticks, *Dermacentor variabilis*, orcokinins was also found to be differentially expressed during female reproduction^60^. **Pyrokinin** is a member of the larger pyrokinin/PBAN peptide family that all share a common C-terminus pentapeptide, FXPRLamide^28^. A variety of physiological functions of pyrokinin/PBAN peptide family have been described, which include activation of sex pheromone biosynthesis and regulation of egg diapause^61^. In this study, the highest expression of pyrokinin was found at the early vitellogenic stage, suggesting that it may be involved in the initialing of vitellogenesis (Fig. 8). **SIF** occur widely in both insects and crustaceans^62^. RNAi-mediated knockdown of SIF or ablation of the SIF expressing cells in the fruit fly, *D. melanogaster* led to alterations in courtship behavior, such as males performed vigorous and indiscriminant courtship directed at either sex while females appeared sexually hyper-receptive^63^. In the giant freshwater prawn, *M. rosenbergii*, *in vivo* injections of SIF enhanced dominance and aggression, which also involved in changes in sexual behavior^64^. In the present study, however, SIF was significantly up-regulated at the early vitellogenic stage (Fig. 8), which suggests that SIF may also be involved in regulating vitellogenesis. Finally, a new peptide **WXXXRamide** was first predicted in a crustacean by the present study, its expression significantly up-regulated at the early vitellogenic stage but decreased sharply at the late vitellogenic stage in *S. paramamosain* (Fig. 8). In addition to the nervous system, *wxxxr* also had a relative weak expression in the ovary tissue of *S. paramamosain* (Fig. 7). Clearly, whether the newly identified neuropeptide in crustaceans playing a role in crustacean reproduction warrants further research.

Although 100 distinct *S. paramamosain* peptides representing 24 neuropeptide families were discovered in this study, a number of well-known crustacean peptide groups were not identified via transcriptome mining, which include bursicon α, bursicon β, corazonin, ecdysis-triggering hormone (ETH), enkephalin, proctolin and RYamide families. Given that only a single organ was used for the present sequencing, it is reasonable to expect that some of the neuropeptide groups would be missed in our transcriptome data. To fully explore the potential neuropeptide groups in *S. paramamosain*, a more comprehensive transcriptome should be studied, and/or a genome for this species should be generated. Clearly, for fully understanding the roles of neuropeptides in *S. paramamosain* reproduction, information on the expression profiles of neuropeptide genes are the first step but far from enough, much work remains to be done. Nevertheless, mining of neuropeptide genes and their differential expression during vitellogenesis of *S. paramamosain* by the present study provides an important basis for subsequent studies on peptide bioactivity in reproduction of the crab species.

## Methods

### Animals

The mud crab ovarian development was classified into the pre-vitellogenic stage, early vitellogenic stage and late vitellogenic stage, according to the morphological and histological characteristics^35^. Six female crabs of each stage were purchased from a local fish market in Xiamen, Fujian Province, China. Prior to dissections, crabs were anesthetized on ice for 30 min. Our study does not involve endangered or protected species.

### Sample preparation and Illumina sequencing

Total RNA from the cerebral ganglia of each stage were isolated separately with the Trizol Reagent (Invitrogen), according to the manufacturer’s instructions, followed by next generation sequencing by BMK (BeiJing Co. Ltd) as per manufacturer’s protocol (Illumina, San Diego, CA). Briefly, poly (A) mRNA was isolated using oligo (dT) beads. Short mRNA fragments were used as templates to synthesize the first-strand cDNA with random hexamers. The second-strand cDNA was synthesized using buffer, dNTPs, RNase H and DNA polymerase I. The short fragments were further purified using a QiaQuick PCR extraction kit and resolved with EB buffer for end reparation, adding of poly (A) and ligation with Illumina Paired-end adapters. After agarose gel electrophoresis, suitable fragments were selected for PCR amplification as templates. A mixed cDNA sample representing three vitellogenic stages in *S. paramamosain* was prepared and sequenced using the Illumina HiSeqTM 2000. Overall, at least 4 Gb of cleaned data (at least 20 million reads) was generated for each of the three samples sequenced. All three samples cycleQ20 percentage was 100%.

### Bioinformatics analyses

Cleaning of low quality reads, assembly, annotation and ORF prediction were done by BMK, using unpublished algorithms (BMK, Beijing Co. Ltd), Trinity^65^, Blast2GO^66^ and Getorf^33^, respectively. For searching neuropeptides what we interest in *S. paramamosain*, the list of annotated sequences and ORF file were scanned for keywords of previously known neuropeptides such as “A-type allatostatin” and conserved amino acid sequences such as “FGLGKR”, respectively. The sequences were re-validated by BLAST. The structures of mature peptides were predicted using a well-established workflow^26^,^27^. Briefly, prohormone cleavage sites were identified based on the information presented in Veenstra (2001)^67^ and/or homology to known propeptide processing schemes. Each of the deduced precursors was assessed for the presence of a signal peptide using the online program SignalP 4.1 (http://www.cbs.dtu.dk/services/SignalP/). Post-translational modifications, e.g. cyclization of N-terminal Gln/Glu residues (Q/E) and C-terminal amidation at Gly residues (G), were predicted by homology to the known peptide isoforms. Schematic diagrams of protein domain structures were prepared using the Domain Graph (DOG, version 2.0) software^68^. Multiple sequence alignment of the predicted peptide sequences was performed with ClustalX. Then the multiple sequence alignment file was exported to LaTEX TexShade^69^ for highlighting the conserved sequence motifs and showing the LOGO.

### Tissue distribution by RT-PCR

Complementary DNA samples derived from ten tissues (see Fig. 7) dissected from female crabs (n = 3) were used for PCR amplification using neuropeptide genes primers (Supplementary Table S2). PCR reactions were performed following a routine protocol optimized for individual neuropeptide genes: 94 °C for 2 min, followed by 32–36 cycles of 94 °C for 30 s, 55 °C for 30 s and 72 °C for 30 s. The house-keeping gene β-actin was concurrently carried out as the internal control, the primer sequence of β-actin gene reference Huang *et al.*^29^. PCR products were analyzed by agarose gel electrophoresis.

### Expression levels verification by quantitative real-time PCR

The qRT-PCR used a 7500 Fast Real-Time PCR (Applied Biosystems) with SYBR Premix Ex Taq (TaKaRa, Japan), and β-actin was used as internal standard gene. Primers for all neuropeptide genes were listed in Supplementary Table S2. PCR reactions were performed as follows: 95 °C for 30 s, followed by 40 cycles of 95 °C for 5s, 55 °C for 30 s and 72 °C for 30 s. *Ct* values were represented by the mean values of five independent replicates, and the relative gene expression levels were calculated using the 2^−ΔΔCt^ method^70^. Five replicate samples of each stage were performed, and the qRT-PCR was repeated three times for each individual sample. All data were expressed as mean ± SD representing the relative expression ratio. The significance of differences in expression was determined using one-way ANOVA followed by Duncan’s multiple range tests. All statistical analyses were performed using SPSS 13.0.

## Additional Information

**How to cite this article**: Bao, C. *et al.* Neuropeptides in the cerebral ganglia of the mud crab, *Scylla paramamosain*: transcriptomic analysis and expression profiles during vitellogenesis. *Sci. Rep.* **5**, 17055; doi: 10.1038/srep17055 (2015).

## Supplementary Material

Supplementary File

## Figures and Tables

**Table 1:** Putative neuropeptide precursors in the cerebral ganglia transcriptome of *S. paramamosain*.

Peptide families	Accession Num.	Size(bp)	Size(aa)	Best Blastx Match			
				Species name	*E*-value	Ident	Accession Num.
Allatostatins							
A-type allatostatin1	KR078370	969	173	*Panulirus interruptus*	5E-26	56%	BAF64528.1
A-type allatostatin2	KR078369	260	86	*Procambarus clarkii*	8E-26	75%	BAE45266.1
B-type allatostatin	KR078384	1865	314	*Pandalopsis japonica*	1E-52	56%	AFV91538.1
C-type allatostatin	KR078363	797	149	*Pandalopsis japonica*	2E-22	48%	AFV91540.1
Prohormone-1	KR078383	559	110	*Bombus impatiens*	2E-20	44%	XP_003488230.1
RFamide peptides							
Neuropeptide F1	KR078371	666	102	*Aplysia californica*	1E-07	56%	NP_001191635.1
Neuropeptide F2	KR078372	673	124	*Litopenaeus vannamei*	5E-50	73%	AEC12205.1
short Neuropeptide F	KR078359	1522	126	*Drosophila ananassae*	9E-06	33%	XP_001962432.1
FLRFamide	KR078378	1888	335	*Procambarus clarkii*	2E-28	38%	BAE06262.1
Myosuppressin	KR078365	685	100	*Procambarus clarkii*	2E-35	79%	BAG68789.1
Sulfakinin	KR078382	485	127	*Homarus americanus*	2E-21	52%	ABQ95346.1
Neuroparsins							
Neuroparsin1	KR078355	1869	101	*Metapenaeus ensis*	7E-16	48%	AHX39208.1
Neuroparsin2	KR078373	1496	106	*Jasus lalandii*	1E-26	65%	AHG98659.1
Neuroparsin3	KR078374	843	97	*Metapenaeus ensis*	4E-12	42%	AHX39208.1
Neuroparsin4	KR078356	2427	102	*Metapenaeus ensis*	2E-16	47%	AHX39208.1
Crustacean hyperglycemic hormones (CHHs)							
CHH1	KR078367	1189	127	*Scylla olivacea*	4E-14	45%	AAQ75760.1
CHH2	KR078357	2329	139	*Scylla paramamosain*	3E-97	99%	AFH36335.1
Orcokinins							
Orcokinin1	KR078380	1672	121	*Procambarus clarkii*	8E-40	64%	Q9NL83.1
Orcokinin2	KR078364	2970	138	*Procambarus clarkii*	4E-70	90%	Q9NL83.1
CCAP	KR078362	507	138	*Callinectes sapidus*	9E-83	88%	ABB46290.1
Diuretic hormone 31	KR078358	699	146	*Homarus americanus*	3E-50	76%	ACX46386.1
Eclosion hormone	KR078366	596	82	*Diaphorina citri*	3E-18	47%	XP_008482994.1
GSEFLamide	KR078375	575	198	*Daphnia pulex*	1E-13	45%	EFX70415.1
HIGSLYRamide	KR078386	979	326	*Apis dorsata*	0.009	27%	XP_006611496.1
Kinin	KR078379	1028	310	*Rhodnius prolixus*	1E-17	36%	DAA34788.1
Pyrokinin	KR078360	1417	342	*Aplysia californica*	4E-11	50%	NP_001191649.1
PDH	KR078368	581	78	*Callinectes sapidus*	2E-46	99%	Q23755.1
RPCH	KR078381	628	108	*Scylla paramamosain*	8E-68	100%	AGW45011.1
SIFamide	KR078361	1567	78	*Cancer borealis*	1E-34	94%	ADO00265.1
Tachykinin	KR078376	926	229	*Panulirus interruptus*	2E-41	43%	BAD06363.1
WXXXRamide	KR078377	1479	375	*Danaus plexippus*	4E-06	27%	EHJ74348.1
Vasotocin-neurophysin	KR078385	364	104	*Acanthisitta chloris*	1E-14	40%	XP_009072277.1
