# Supplementary material for: Neuropeptides in the cerebral ganglia of the mud crab, Scylla paramamosain: transcriptomic analysis and expression profiles during vitellogenesis
Source: Sci Rep. 2015 Nov 23;5:17055. doi: 10.1038/srep17055 (PMC4655400; doi:10.1038/srep17055)
Supplement: Supplementary File [file srep17055-s1.pdf]

Neuropeptides in the cerebral ganglia of the mud crab, *Scylla paramamosain*: transcriptomic analysis and expression profiles during vitellogenesis

Chenchang Bao[1], Yanan Yang[3], Huiyang Huang[1], Haihui Ye[1,2]*

[1]College of Ocean and Earth Sciences, Xiamen University, Xiamen 361102, China
[2]Collaborative Innovation Center for Development and Utilization of Marine Biological Resources, Xiamen 361102, China
[3]State Key Laboratory of Marine Environmental Science, Xiamen University, Xiamen 361102, China
∗Correspondence: haihuiye@xmu.edu.cn

Supplementary file

A-type allatostatin1 (AST-A1)

+VVVVVVVGLPPPTMAQLGDYDLQYDPNTLLQILQQYEAAAAEAVAVAEAEAAAAEELEDEENYSYGKRQTPDHYSFGLGKRTPQPYAFGLGKRGGVYSFGLGKKSDVYSFGLGKKSGNYNFGLGKRSVREVASEEEEEVKKELQEDKEGEAKRTKRDVNDEDKEEEEKEGCE

A-type allatostatin2 (AST-A2)

+YEFGLGKRGGLYEFGLGKRGSGQYAFGLGKKAGQYSFGLGKRDQGDASDSYTLGRRSGSYSFGLGKRGGPYSFGLGKRDGPYSFGL+

B-type allatostatin (AST-B)

MQLATLTATLLTLMAAAAAQDEGSGLAVAQAKRAGWSSMRGAWGKRDDSSDQGLQVSEDKRNNNWRKFQGSWGKRGEEIQDAEEKRGNWNKFQGSWGKRADDMTEEAALQAAEDKRAGWNKFQGSWGKRGDEVASEDDLQDAEDKRTSWGKFQGSWGKRQDDLIQLQDLEDKRNNWSKFQGSWGKRAGWSSLQGAWGKRAWSNLQGAWGKRSPNDSEDIEDEALEEEELQVSPEALARMVAASPVKRGWALWGKRPDYPAVSPRSTNWSSLRGTWGKRSGDWSSLRGAWGKRVPNDWAHFRGSWGKRSPDTLIA

C-type allatostatin (AST-C)

MMPCPGHLVVALALVLTLSHALPAKEVPKAQKEVSSAHEGGRLQKRAAGPSSDTSQEELEALKDLILSRLAAELDATYQDLPSFKHDLLKAEVDGEEDDEGNEEGRREEGKKKRMFAPLSGLPGNLRTIKRQIRYHQCYFNPISCFRRK

Prohormone-1

MSPRLSTVLILAVVVLAALGTTSAKPLGEQDPSAGGPPAFTAREAQVYEPYGNNLEEDGSLDAALINYLFAKQLVQRLRSPSEVSRESQRKRSYWKQCAFNAVSCFGKRK

Neuropeptide F1 (NPF1)

MRGTLTVVAVVVVSVAAAWAAQLPSRQEGGALDAIQALHEAALAGTLSLGEVPYPSRPNVFKSKGELRRYLDAINAYFAIAGRPRFGKRGEQVRQPEELYDY

Neuropeptide F2 (NPF2)

MCRQLLTALVVGVVVVGALEMGGAEGKPDPTQLAAMADAIKYLHELDKYYSPVSRPSTRSAPGPASHIQALEKTLKFLQLQELGKIYSHMTRPRFGKRSETVLPPGDTLMEASERLLETLARRR

Short neuropeptide F (sNPF)

MGVNGVKCWVALVCCCLLLCQLTTATPVDYDTLNDMYDLLAVHEVERRAPPSMRLRFGKRDMGWQVAQRSMPTLRLRFGKRNVDEADPILDHHDLIRKDARTPALRLRFGKRGASFGEEDMVSQEQ

FLRFamide

MIAVAWVLLSGVAWCLASPLTPVPGAIEASPSTHNAPGSDIPEIPQEKRLLKYFLPSSSSWMPTQQEGSKRGYSKNYLRFGRSEEDKRGGRNFLRFGRADISSIEDTDMLPETEDSLEKRNRNFLRFGRDRNFLRFGRSDAEEFGLPGGPLAFSNLQEDDTEDYPVEEKRSGHRNYLRFGRGNRNFLRFGRDDSRNFLRFGRSVDRQLKEQKAHEAPLAPTVVPHSPAKTQDSHRSKRSASPYNYVVMPSHGPAAWAQDFQPDQEDEDLDMAIDGPEGAVSKRGYNRSFLRFGRDRNFLRFGKRNDDSASDVVVVEPASYPRYQRAPQRNFLRFG

Myosuppressin

MVFRLQPWCSLLLVGVVVVLGVCAGVGETIPPPICFNQKLVLTPYARRLCAALNDISKFSRAMEDYLDAQAIKNSMGVNEPEVKRQDLDHVFLRFGRAQQ

Sulfakinin

+GEGGSLPARPLPQRPAAALARVLAPVVRHRLEEEEEGLLEELLEEEGGVVGGAGPVEVLDAAGKREFDDYGHMRFGKRGSGNDDYQDDYGHLRFGRSYHHTTHRHNNNNNNYHHRRFNSLQNGSAKV

Neuroparsin1 (NP1)

MASFCCRATTLVLVCSCLLLLLQEASGAPRCDKHDQEAPKNCRYGTTLDWCKNGVCAKGPGETCGGYRWSEGKCGEGTFCSCGICGGCSPFDGKCGPTSIC

Neuroparsin2 (NP2)

MEMTTRSYIFFFIVSSTALLLLLPGRCEGGPICSSLNEVLPEMLQAPCRHGVVMDWCGNARCAKGPGETCGGRWNVKGSCGKGMYCVCGYCAGCSWDLQCALGRFC

Neuroparsin3 (NP3)

MDNSRKAPYLVMIVSLLFFPSNVFSTPLCSSDNEVQPSECPHGTVTNRCGNTVCAKGLREPCMVYRWERDLCGGGTFCGCGFCMGCNNNLQCWDCGS

Neuroparsin4 (NP4)

MTPRARPATLILASCLLLLLLLLQKGSAIPRCSVYDQPPPKNCKYGTALDWCSNGVCAKGPGETCGGYRREEGICGEGTYCECGRCRGCSPFDASCHDAQLC

Crustacean hyperglycemic hormone1 (CHH1)

MFDKITTLAVVMVVVIVIALNIDGGAAGPVKPRHNPVSTGGDAGQQHRRSRRATDLVDTSCKGYYDRDTWNELHHLCEDCDNLYRQFHFQAKCRSDCFASDVFATCLTDLGKNVEIYQAMAASLRGS

Crustacean hyperglycemic hormone 2 (CHH2)

MSTFTSVIQMAVLVACIAMATLPHTQGRSADGFGRMGRLLASLKADSLGPVQDYGVEGAAHPLEKRQIFDSSCKGVYDRAIFSELEHVCNDCYNLYRTSRVASGCRANCFENHVFDDCVYDLLLHNPDEVLLMRDAIRG

Orcokinin1

MTRDVFCTALLLALCVMASEGAIKDAPAHANNHPDAGYPSDGSSAKRFDAFTTGFGHSKRNFDEIDRSSFGFAKRNFDEIDRSSFGFVKRMLTPRDLANLYKRNFDEIDRSGFGFVRRNAE

Orcokinin2

+KRNFDEIDRSGFGFAKRNFDEIDRSGFGFAKRNFDEIDRSSFGFNKRNFDEIDRSGFGFAKRNFDEIDRSGFGFAKRNFDEIDRSSFGFNKRNFDEIDRSSFGFVKRMLTPRDLANLYKRNFDEIDRSGFGFVRRNAE

Crustacean cardioactive peptide (CCAP)

+TSLSGRAGLVTAATILLLAFLAADTAAGPVAKRDIDSLLDGKIKRPFCNAFTGCGKKRSDPELEGLASGSELNDITKHVLAEARLWEQLQNKMEAMRMLASRMESRPMFRRKRSLTQPQHDHVHSAAALEHKGDVEKQ

Diuretic hormone 31 (DH31)

MNNFGVVFASLVAAFILLSSVHATPINREPSRAVVEIDDPDYVLELLTRFSNSIIRAKELEKFVRSSSGTKRGLDLGLGRGFSGSQAAKHLMGLAAANYAGGPGRRKRESQAAPLALHHDDHPAAQEHAAVAADAAAAGLQQHSSR

Eclosion hormone (EH)

MSLKPEVRAVVLGLVCLVVLATVGEAATIIGMCISNCGQCKEMYGDYFHGQACAESCIRTHGVTIPDCNNPATFNRFLKRFI

GSEFLamide

MVQGTPCLTKCIVVLSCVSCVLSAALQNTQPDESQSDVEKRAARDPMLRYLLVAMAQPGPRYAAPQILSRGVRRIGSEFLGKRSIAVSGTEKMCEPDPCVAEEDRDHADDLKKEQMSFTGQYNEQNGAGDLQDAPDKRALGDLSRSRLARYFSLLLNKKMGSEFLGKRAMGSEFLGKRAMGSEFLGKRAMGSEFLGKR+

HIGSLYRamide

+AAPTPAPAGPDAAQHQEEPAREKRFLGSLLRQDGRLPIWGYKRSFIGQQDSNSDHDVMTPAAASPAEGTLPEDSEAQALEKRHLSSLARNNAFPKDLREKKYFASLLKSRVMGEGTKFSQQDAPDDSESRLQKRFYASLLKSDTPPQTAYLNSVFYRQDKRHYGSLLRSGPLPFMQDKRHFASLLKSPSYRGISIPGKRGVGAEDGAQDDAVRQLEDVRELSKHQFSSLLGNHLDDDLELQRRLLAAGLISPGDTQDLAALFTPWEVATLPGQDKRHIGSLYRGKKDEDSLYDLPEDKRHIGSLYRGKKDEDSLYHELSEDKRHIG+

Kinin

+PHTQVDSYRQESRRPRFLPWGGKRSGMERGKFEDVLHAWATEGDSTAWPASIQMNADMKRKAFSAWAGKRNGDNKRGGFSAWAGKREAFGPWKGKRSEEDEKRQAFSAWAGKRSEDEKRQAFSAWAGKREAFNAWAGKRSSNDNDKRQAFSAWAGKRSNNDDEKRKPFSAWAGKRSDENNDKRQAFNAWAGKRSNSDYDKRQAFSAWAGKRSNNDNKRQGFSAWAGKRSNNEDKRQGFSAWAGKRKFNAWAGKRSDDDYYQEQEEKEEEKSRDQLSSLLQQHHQQEHQQASSLLQHSPDSLTHWDANWDR

Pyrokinin

MLLNPTKLLVPAPLVCLALISTCVSLVAGATEGAVSSPEWSPPPVPPRHLGPPAPWEHVASDAVSSLLAGPINAQNIMRLQRIPERTSRPLKIMTPGVPKRLYFAPRLGKRSPSLVESLDERGRRDAASHNEDLEDAVTIPYSWWWPLVSVRRSSFSPRPGKRAEGEEMDLPYDYYDSEDDEDEETEDEDEDEDEGVLQDKRDSAFAFSPRLGKRVQKSAFAFAPRPGKRSNFAFAPRPGKRTGSIFAFAPRPGKRTNFAFAPRPGKKSNFAFAPRPGKKTSFAFSPRLGKKADFAFAPRPGKRSDSPSETEDRQTGETWWIGEGASSTVTTQPPFLPPRLE

Pigment-dispersing hormone (PDH)

MRSAVIVAVLVVVALAALLTQGQELKYQEREMVAELAQQIYRVAQAPWAAAVGPHKRNSELINSILGLPKVMNDAGRR

Red pigment-concentrating hormone (RPCH)

MVRRAGVTLLVVALVVVALMSSVSAQLNFSPGWGKRAAATSSSNGGVGEGVSGLHPSVGGAPGGVVPPGSSSGDSCGPIPVSAVMHIYRLIRSEAVRLVQCQDEEYLG

SIFamide

MSMQMRVVVAVAVVVVVLALLSSPVSAGYRKPPFNGSIFGKRSGSDAVYEPGKSQALASVCQVALEACTLWFPGAEKK

Tachykinin

MSRMWVWAAMVVVGVAAVAAASGAGQGEAGSDAPRPRRAPSGFLGMRGKKEAPSPPLQDAPAPPEDLLPALYQLDVP

LRGKKTPSGFLGMRGKKSEEEEEEDEETPFARPSYESEFDNLVKRYPSGFLGMRGKKTPSGFLGMRGKKSSGEDYLPSTS
ISRQALLSLLQGDAAPPKDEDYYYYYYNPEAWRAGTHKRAPSGFLGMRGKKDAYPGLAQDKRTPSGFLGMRG

WXXXRamide

MASVFGLLVMALAAALAATAQELSPGEAGSSEGQAWEGHRHARSLETGGDASWLSGLQDADVTQVEPRVEAGHGGTT
FWVARGKKDAENSPTYYWGPNQGLWGETFQRGTSATASSSHTPLAVKSGWESNPSLWGKRDGRGPFWAARGKRPDPF
WASRGRRDSSEMTSALLQQWAAEDPTMQEQKEDQLWSGEIKREEGGPFWISRGKRPQPGSAASQLASLWAIRGRKSGA
DNTFWAARGKKETNVRGPFWAARGKRSGGEGGTGPYWIARGKKQDGNTPTGPYWIARGKKEDDGGVFWAARGKKD
PPAWATGRGRREDAANSFWIARGKKSEHAASHNTEKDNDDDDDEEQHEEVTQKAADHYLKGFTALSGK

Vasotocin-neurophysin

+GRCVGPDICCGARIGCFLGSRETRMCRTENMVPITCYNSDLKPCGRMQEGRCGAPGICCTENKCEMNDDCVAEDTQG
EEVEESQRGGRPRLDLLAAARDRWEEQ

Table S1 | Mature peptides for neuropeptide families	
**Family**	**Predicted mature peptide structure**
**AST-A**	QTPDHYSFGLa
	TPQPYAFGLa
	GGVYSFGLa
	SDVYSFGLa
	SGNYNFGLa
	+YEFGLa
	GGLYEFGLa
	GSGQYAFGLa
	AGQYSFGLa
	SGSYSFGLa
	GGPYSFGLa
	DGPYSFGL+
**AST-B**	AGWSSMRGAWa
	NNNWRKFQGSWa
	GNWNKFQGSWa
	AGWNKFQGSWa
	TSWGKFQGSWa
	NNWSKFQGSWa
	AGWSSLQGAWa
	AWSNLQGAWa
	GWALWa
	TNWSSLRGTWa
	SGDWSSLRGAWa
	VPNDWAHFRGSWa
**AST-C**	QIRYHQCYFNPISCF
**Prohormone-1**	SYWKQCAFNAVSCFa
**NPF1**	AQLPSRQEGGALDAIQALHEAALAGTLSLGEVPYPSRPNVFKSKGELRRYLDAINAYFAIAGRPRFa
**NPF2**	KPD
	PTQLAAMADAIKYLHELDKYYSPVSRPSTRSAPGPASHIQALEKTLKFLQLQELGKIYSHMTRPRFa
**sNPF**	APPSMRLRFa
	SMPTLRLRFa
	KDARTPALRLRFa
**FLRFamide**	GYSKNYLRFa
	GGRNFLRFa
	NRNFLRFa
	(2x) DRNFLRFa
	SGHRNYLRFa
	GNRNFLRFa
	DDSRNFLRFa
	GYNRSFLRFa
	APQRNFLRFa
**Myosuppressin**	pQDLDHVFLRFa

**Sulfakinin**	EFDDYGHMRFa<br>GSGNDDYQDDYGHLRFa
**NP1**	APRCDKHDQEAPKNCRYGTTLDWCKNGVCAKGPGETCGGYRWSEGKCGEGTFCSCGICGGCSPFDGKCGPTSIC
**NP2**	GPICSSLNEVLPEMLQAPCRHGVVMDWCGNARCAKGPGETCGGRWNVKGSCGKGMYCVCGYCAGCSWDLQCALGRFC
**NP3**	TPLCSSDNEVQPSECPHGTVTNRCGNTVCAKGLREPCMVYRWERDLCGGGTFCGCGFCMGCNNNLQCWDCGS
**NP4**	IPRCSVYDQPPPKNCKYGTALDWCSNGVCAKGPGETCGGYRREEGICGEGTYCECGRCRGCSPFDASCHDAQLC
**CHH1**	ATDLVDTSCKGYYDRDTWNELHHLCEDCDNLYRQFHFQAKCRSDCFASDVFATCLTDLGKNVEIYQAMAASLRGS
**CHH2**	QIFDSSCKGVYDRAIFSELEHVCNDCYNLYRTSRVASGCRANCFENHVFDDCVYDLLLHNPDEVLLMRDAIRG
**Orcokinin**	FDAFTTGFGHS<br>NFDEIDRSSFGFA<br>(2x) NFDEIDRSSFGFV<br>(2x) NFDEIDRSGFGFV<br>(4x) NFDEIDRSGFGFA<br>(2x) NFDEIDRSSFGFN
**CCAP**	PFCNAFTGCa
**DH31**	GLDLGLGRGFSGSQAAKHLMGLAAANYAGGPa
**EH**	ATIIGMCISNCGQCKEMYGDYFHGQACAESCIRTHGVTIPDCNNPATFNRFL
**GSEFLamide**	IGSEFLa<br>MGSEFLa<br>(3x) AMGSEFLa
**HIGSLYRamide**	(2x) HIGSLYRa<br>HIG+
**Kinin**	PRFLPWGa<br>KAFSAWAa<br>GGFSAWAa<br>EAFGPWKa<br>(4x) QAFSAWAa<br>EAFNAWAa<br>KPFSAWAa<br>QAFNAWAa<br>(2x) QGFSAWAa<br>KFNAWAa
**Pyrokinin**	LYFAPRLa<br>SSFSPRPa<br>DSAFAFSPRLa<br>VQKSAFAFAPRPa<br>(2x) SNFAFAPRPa<br>TGSIFAFAPRPa

	TNFAFAPRPa
	TSFAFSPRLa
	ADFAFAPRPa
**PDH**	NSELINSILGLPKVMNDAa
**RPCH**	pQLNFSPGWa
**SIFamide**	GYRKPPFNGSIFa
**Tachykinin**	(2x) APSGFLGMRa
	(3x) TPSGFLGMRa
	YPSGFLGMRa
**WXXXRamide**	VEAGHGGTTFWVARa
	DGRGPFWAARa
	PDPFWASRa
	EEGGPFWISRa
	PQPGSAASQLASLWAIRa
	SGADNTFWAARa
	ETNVRGPFWAARa
	SGGEGGTGPYWIARa
	QDGNTPTGPYWIARa
	EDDGGVFWAARa
	DPPAWATGRa
	EDAANSFWIARa

Table S2 | Primers for neuropeptide genes used for qRT-PCR

Neuropeptide genes	Primer sequence (5’-3’)
**AST-A1**	**F**-AAGCGACAAACTCCTGACCAT
	**R**-GCCAAAGTTGTAGTTCCCAGAT
**AST-B**	**F**-CCTCTTCTGCGAGCAACACTT
	**R**-GACGCCATCCACTTCATGTAGTA
**AST-C**	**F**-TCACTCGCTGCTTGTTATCCT
	**R**-AACCCCATCTCCTGCTTCA
**Prohormone-1**	**F**-TCCCTGACGCCCCTGCTA
	**R**-CCCATCCTCCTCAAGATTGTT
**NPF1**	**F**-GAAGCGAGGCGAGCAAGTA
	**R**-GGTGAGGGTGGTGAGGGAG
**NPF2**	**F**-AGAGGCTGCTGGAGACACTGG
	**R**-AATGAGACAAAGACCGAACACCT
**sNPF**	**F**-GGTGACTCCGATTTAATGCTTT
	**R**-TGGCTTCCACTGCCGCTA
**FLRFamide**	**F**-ACGATACTGAGGACTACCCTGTGG
	**R**-TGCTTCATGGGCCTTCTGTTC
**NP1**	**F**-CCACCAGGATTTCACCCGTT
	**R**-GGAGGAGCAGGAGGCAAGA
**NP2**	**F**-TTGTAATGGACTGGTGCGGTAA
	**R**-GGAGGAGTAGGTAAGCGAGGA
**NP3**	**F**-CCCTCCGCCCTTGTACTACTCTA
	**R**-TGTTTCTGGTGATGGGTAACGAA
**NP4**	**F**-CAGTGATACGGCGTCTAACCTC
	**R**-GGCGAGGCTTATCTGACAATC
**CHH1**	**F**-TCACTGATTTGGGCAAGAACG
	**R**-CTCGGCATGTCACTAGAGGGT
**CHH2**	**F**-CTACAATCTTTACAGAACATCCCGT
	**R**-TCATAAGCAGCACCTCGTCAG
**Orcokinin2**	**F**-CATCCCAAATACCAGAAGTCCA
	**R**-GCTTGAGTGCTGTGCTAGAAACA
**CCAP**	**F**-CGAGGCAAGGTTATGGGAG
	**R**-GATACACAGAGCCACTCAAGAAAT
**DH31**	**F**-TCCTCGCCATTCTCCTCACA
	**R**-GGGTCGTCTATCTCCACTACAGC
**Pyrokinin**	**F**-CCGTCACCATCCCCTACAG
	**R**-GAGAACGCAAATGCCGAGT
**PDH**	**F**-CTCGCAGCCCTACTCACCCA
	**R**-CGGCGTCGTTCATCACCTT
**SIF**	**F**-CTGGAGCCGAGAAGAAATGAA
	**R**-ATACAGCGGTGGTGATGGTG
**WXXXRamide**	**F**-CGCAGAGAACTCCCCAACTTAC
	**R**-GCAGCAGGGCAGACGTCA
